# Several Common Genetic Variations Associate With Functional or Anatomic Effects of Anti-VEGF Treatment in Conditions With Macular Edema

**DOI:** 10.1167/iovs.66.6.2

**Published:** 2025-06-02

**Authors:** Ingeborg Klaassen, Maartje J. C. Vader, Khadija Aissa, Michael W. T. Tanck, Reinier O. Schlingemann, for the BRAMD, BRVO, and BRDME Study Groups

**Affiliations:** 1 Ocular Angiogenesis Group, Department of Ophthalmology, Amsterdam UMC, University of Amsterdam, Amsterdam, The Netherlands; 2 Microcirculation, Amsterdam Cardiovascular Sciences, Amsterdam, The Netherlands; 3 Cellular & Molecular Mechanisms, Amsterdam Neuroscience, Amsterdam, The Netherlands; 4 Department of Epidemiology and Data Science, Amsterdam UMC, University of Amsterdam, Amsterdam, The Netherlands; 5 Department of Ophthalmology, University of Lausanne, Jules-Gonin Eye Hospital, Lausanne, Switzerland

**Keywords:** diabetic retinopathy, branch retinal vein occlusion, age-related macular degeneration, anti-vascular endothelial growth factor, genome-wide association

## Abstract

**Purpose:**

This study aimed to identify genetic factors that may influence the effectiveness of anti-vascular endothelial growth factor (VEGF) treatment for macular edema (ME).

**Methods:**

We performed a genome-wide association study (GWAS) on 606 patients with macular edema that were treated with bevacizumab or ranibizumab from three randomized clinical trials, using a Infinium Global Screening Array. Well-characterized patient groups included diabetic macular edema (DME), retinal vein occlusion (RVO), and neovascular age-related macular degeneration (nAMD), with changes in best-corrected visual acuity (BCVA) and anatomical changes in central subfield thickness (CST) as outcomes. We conducted a meta-analysis on the combined patient groups, a targeted analysis on 28 previously reported genetic variants related to anti-VEGF response, and on variants of six genes involved in ME pathophysiology.

**Results:**

GWAS meta-analysis identified 12 SNPs (*P* < 1 × 10^−6^), of which three SNPs reached genome-wide significance (*P* < 5 × 10^−8^) and were linked to changes in CST after 6 months of anti-VEGF treatment. In addition, a genomic locus in the *SGCZ* gene was linked to changes in BCVA. Targeted GWAS revealed significant associations between changes in BCVA and variants in *KDR*, *IL6*, *NRP1*, and *SPNS2*, all known for their roles in VEGF signaling and retinal vascular permeability. Furthermore, changes in CST were associated with *PLVAP*, an important gene in vascular hyperpermeability in ME.

**Conclusions:**

This GWAS meta-analysis uncovered genetic variants potentially linked to responses to anti-VEGF treatment in patients with retinal conditions featuring vascular leakage and/or ME. These findings could aid in identifying genetic markers for treatment response prediction or uncover new pathways relevant to retinal vascular leakage and ME.

The most common retinal vascular disorders that can cause vision loss through the sequelae of macular edema (ME) are diabetic retinopathy (DR) and retinal vein occlusion (RVO). In addition, in neovascular age-related macular degeneration (nAMD), retinal swelling and cystic macular edema are almost invariably present. In each of these disorders, breakdown of the inner endothelial blood–retinal barrier (BRB) results in the extravasation of proteins, lipids, and fluid into the retina that exceeds clearance kinetics, causing macular edema, damage to neurons, and loss of vision.^1^

Despite ongoing research in this field, the understanding of the molecular mechanisms causing macular edema is still incomplete. To develop alternative therapies beyond those targeting vascular endothelial growth factor A (VEGFA) and to identify predictive biomarkers for anti-VEGF responses, it is essential to explore novel pathways.

Currently, the most effective treatment option for ME is intravitreal injection of anti-VEGF drugs. Although this treatment has resulted in dramatic improvements in best-corrected visual acuity (BCVA) and a decrease in central subfield thickness (CST) of the retina for many patients, it represents a true burden for the patients and has high costs. Also, a high degree of variability in treatment response has been reported.^2^ Approximately 10% of patients with nAMD show a decline in BCVA of at least 15 letters (i.e., 3 lines) on the Early Treatment Diabetic Retinopathy Study (ETDRS) letter chart despite treatment, and, in patients with diabetic macular edema (DME), up to 50% of patients do not fully respond or are refractive to this therapeutic approach.^3^ In previous studies, several factors have been identified which, to some extent, predict the response to anti-VEGF treatment, such as younger age, prompt treatment initiation, and smaller area of neovascularization in nAMD.^4^ In blood samples obtained from 89 patients with DME according to the study protocol of the Bevacizumab and Ranibizumab in Diabetic Macular Edema (BRDME) study, we found that circulating messenger RNA (mRNA) levels of retinoschisin and rhodopsin are associated with visual acuity and changes in central subfield thickness during anti-VEGF therapy.^5^ However, these associations remain unexplained, and sensitivity and specificity are too low for their use as a predictive biomarker for anti-VEGF responsiveness for individual patients. In addition, we investigated plasma microRNA (miRNA) levels in DME patients but could not demonstrate associations between miRNA levels and the response to anti-VEGF therapy.^6^

As an alternative to mRNA and miRNA, common genetic variations may identify a genetic biomarker profile that could distinguish “worse” responders from “better” responders. Additionally, this approach may help uncover novel pathways underlying macular edema. Using genome-wide association studies (GWASs), researchers have identified a small number of single nucleotide polymorphisms (SNPs) associated with DME^7^ or BCVA outcome in nAMD.^4^ Furthermore, a recent GWAS study involving 220 DME patients has identified SNPs that are related to the response to anti-VEGF treatment in terms of BCVA and central macular thickness (CMT).^8^

In this study, we conducted a GWAS in 606 well-characterized patients with DME, macular edema secondary to RVO, and nAMD treated with bevacizumab or ranibizumab. Our aim was to relate our findings to BCVA and anatomical outcomes from optical coherence tomography (OCT). The unique study design was motivated by the idea that, by including these three different retinal conditions, each with potentially distinct underlying genetic mechanisms, we could specifically investigate their common pathways of VEGF-driven macular edema. Moreover, this design enabled us to validate our findings across the three study groups.

## Methods

### Patient Characteristics

Blood samples for DNA analysis were collected from the BRAMD study, which compared the effectiveness of bevacizumab to ranibizumab in patients with AMD (BRAMD); the BRVO study, which compared the efficacy of bevacizumab to ranibizumab in patients with RVO; and the BRDME study, which compared bevacizumab to ranibizumab in DME; the study protocols have been detailed previously.^9^^–^^11^ In summary, the BRAMD, BRVO, and BRDME trials were prospective, randomized, double-masked clinical trials with a non-inferiority design, performed in eight clinical centers throughout The Netherlands. The Institutional Review Board/Ethics Committee approved the trial protocols, and the studies followed the tenets of the Declaration of Helsinki. All participants signed written informed consent before screening. More details can be found in the Supplementary Materials.

The trials are registered at ClinicalTrials.gov and at The Netherlands Trial Register (NTR1704 for the BRAMD study; NTR3257 for the BRVO study; NCT01635790 and NTR3247 for the BRDME study). During informed consent, a few patients objected against genetic tests. For the present study, blood samples were available from the BRDME trial (*n* = 120), the BRVO trial (*n* = 229), and the BRAMD study (*n* = 257). The genetic studies were approved by the Medical Ethical Review Committee of the Academic Medical Center Amsterdam. The participation of the other centers was reviewed at each center according to Dutch law. The data collected included demographic variables, OCT measurements, BCVA, and comorbidities such as hypertension or diabetes.

### Outcome Measures

As outcome measures of anti-VEGF therapy, we used the change in BCVA and CST at 6 months after treatment onset in all three studies. Change in BCVA was expressed as the difference of ETDRS letters from baseline. Relative CST decreased values were calculated as compared to baseline values, as described in the Supplemental Material.

### Statistical Analyses

Continuous variables are described as median with interquartile range (IQR) and were compared among the patient groups using a Kruskal–Wallis test. In case of a significant difference, pairwise comparisons were made using Mann–Whitney tests. Categorical variables are described as number and percentages and were compared among patient groups using Fisher exact tests. Spearman correlation coefficients were calculated between (baseline) continuous variables in the total set and the separate patient groups.

### Genome-Wide Association Analyses

Details on processing of blood samples, genotyping, imputation, and quality controls are given in the Supplementary Materials. The study includes data derived exclusively from individuals of European ancestry. Association analyses with (empirical normal quantile transformation [ENQT]) changes in CST and BCVA were performed for each of the three patient groups using an additive linear regression model implemented in PLINK 2.0 (Whole-Genome Association Analysis Toolset), correcting for sex and age of the individual, their baseline CST/BCVA, and the first five principal components. The summary results of the three patient groups were then combined using an inverse variance-weighted fixed-effects meta-analysis, performing meta-analysis heterogeneity analysis, implemented in METAL (Meta-Analysis Tool, version 2011-03-25). SNPs that had a minor allele frequency (MAF) < 0.05 in any patient group, as well as those with a heterogeneity test *P* <1 × 10^−^^7^ were excluded. Q-Q and Manhattan plots were generated using the GWASTools^12^ package in R (R Foundation for Statistical Computing, Vienna, Austria). Variants associated with a *P* < 5 × 10^−^^8^ were considered genome-wide significant; the suggested threshold was *P* < 1 × 10^−^^6^. For annotation purposes, meta-GWAS summary statistics were uploaded to the Functional Mapping and Annotation (FUMA) platform.^13^

### Previously Reported SNPs and Targeted Gene Approach

We used targeted gene analysis to refer to hypothesis-driven follow-up of candidate genes or SNPs in our GWAS dataset. Special attention was given to SNPs that have previously been associated with anti-VEGF response (see Table 3).^4^^,^^8^^,^^14^^–^^18^ Additionally, we looked at SNPs in several genes known or presumed to function within the molecular pathways underlying the pathophysiology of macular edema, including *VEGFA*, *VEGFC*, erythropoietin (*EPO*), nitric oxide synthase 3 (*NOS3*, or eNOS), apolipoprotein E (*APOE*), and plasmalemma vesicle-associated protein (*PLVAP*) (see Table 4).^19^^–^^25^

### Power Statement

Given the total sample size of 606, we calculated minimal effect sizes per effect allele that could be detected with 80% power for SNPs with varying MAF assuming a (log)additive genetic model and a (genome-wide) significance threshold of 5 × 10^−8^. For SNPs with MAFs ranging from 0.05 to 0.5, the minimal detectable effect sizes ranged from 0.8 to 0.4. Given the observed standard deviations for (relative) change in BCVA (13 letters) and CST (36%), the minimal detectable changes per effect allele would be 12 to 5 letters for change in BCVA and 32% to 14% for relative change in CST, respectively.

## Results

### Patient Characteristics

Demographic information and clinical parameters for the patient groups are detailed in Table 1. For all patients, there was a median improvement of 9.0 ETDRS letters in BCVA and a median reduction of 138.0 µm in CST after 6 months of anti-VEGF treatment. These changes indicate significant improvements in vision and macular thickness (*P* < 0.05 for both measures). On average, the baseline BCVA was higher in the BRDME group, but the change in BCVA was greater in the BRVO group compared to the other patient groups. Additionally, there were significant differences in baseline CST values among all patient groups, with the highest average values observed in the BRVO group. The change in CST was also higher in the BRVO group compared to the other patient groups (Table 1). The median relative decrease in CST was most significant in the BRVO group (79.9%) and least in the BRDME group (30.0%). Consequently, the percentage of patients with a more than 70% decrease in CST (the high responders) was highest in the BRVO group (63.3%), compared to the percentages in the BRAMD (24.1%) and BRDME (20.0%) groups.

**Table 1.** Demographic and Clinical Characteristics of the Three Study Groups

Characteristic	All	BRDME	BRVO	BRAMD	*P* ^*^
Patients, *n*	606	120	229	257	—
Age at first injection (y), median (IQR)	73.5 (65.0, 80.0)	66.0 (57.8, 72.0)^a^	69.0 (62.0, 77.0)^b^	79.0 (74.0, 82.0)^c^	<0.001
Female, *n* (%)	310 (51.2)	48 (40.0)^a^	121 (52.8)^a,b^	141 (54.9)^b^	0.022
BMI (kg/m^2^), median (IQR)	26.8 (24.5, 29.8)	28.2 (25.20, 31.7)^a^	26.2 (24.2, 29.0)^b^	25.7 (23.4, 29.6)^c^	<0.001
HTN, *n* (%)^†^	110 (18.3)	15 (12.5)	51 (22.3)	44 (17.5)	0.073
DM, type 2, *n* (%)	—	104 (86.7)	0	0	—
HbA1c (mg/dL)^‡^	—	7.6 (1.5)	NA	NA	—
Baseline BCVA (letters), median (IQR)	64.0 (54.0, 72.0)	70.0 (64.0, 75.0)^b^	62.0 (51.0, 72.0)^a^	62.0 (52.0, 71.0)^a^	<0.001
Change in BCVA (letters) after 6 months, median (IQR)^§^	9.0 (3.0, 16.0)	5.0 (1.0, 10.0)^a^	14.0 (8.0, 22.0)^b^	6.0 (0.0, 12.0)^a^	<0.001
Non-responders	20.0 (13.3, 27.0)	14.0 (10.8, 16.0)	28.0 (24.0, 38.0)	16.0 (13.0, 21.0)	
Slow responders	8.0 (5.0, 13.0)	5.0 (4.0, 7.0)	14.0 (12.0, 16.0)	6.0 (4.0, 8.0)	
High responders	−1.0 (−5.8, 2.0)	−1.0 (−3.3, 0.3)	4.0 (−1.0, 7.0)	−5.0 (−8.0, 0.0)	
Baseline CST (µm), median (IQR)	444.0 (357.3, 585.1)	449.0 (404.9, 522.8)^b^	583.4 (446.0, 723.1)^c^	361.0 (311.0, 445.0)^a^	<0.001
Change in CST (µm), median (IQR)^§^	−138.0 (−275.4, −61.3)	−90.6 (−183.8, −31.0)^b^	−268.2 (−414.0, −119.4)^a^	−109.0 (−177.0, −51.0)^b^	<0.001
Non-responders	−363.5 (−501.3, −219.3)	−236.5 (−311.0, −191.8)	−538.0 (−673.0, −439.2)	−227.0 (−346.0, −186.0)	
Slow responders	−133.5 (−220.9, −97.0)	−90.6 (−120.5, −68.5)	−268.2 (−318.7, −211.3)	−109.0 (−131.0, −90.0)	
High responders	−31.0 (−59.0, −6.0)	−1.4 (−24.8, 26.4)	−65.0 (−109.0, −31.0)	−25.0 (−45.0, −13.0)	
Relative change in CST (% decrease, 0%–100%), median (IQR)^§^	60.7 (30.2, 81.2)	30 (8.8, 63.0)^a^	79.9 (60.0, 90.3)^c^	52.5 (30.0, 69.3)^b^	<0.001
Non-responders	13.2 (2.4, 21.3)	8.8 (1.9, 18.4)	5.8 (0.0, 19.4)	17.8 (8.0, 25.8)	
Slow responders	54.6 (43.4, 62.4)	52.7 (41.7, 60.2)	58.3 (45.6, 64.1)	53.1 (42.9, 61.7)	
High responders	84.5 (79.0, 92.8)	84.3 (75.9, 89.2)	84.3 (75.9, 89.3)	80.8 (76.5, 87.5)	
Relative change in CST, *n* (%)^§^					<0.001
Non-responders	150 (24.7)	60 (50.0)	25 (10.9)	65 (25.3)	
Slow responders	225 (37.1)	36 (30.0)	59 (25.8)	130 (50.6)	
High responders	231 (38.1)	24 (20.0)	145 (63.3)	62 (24.1)	

BMI, body mass index; DM, diabetes mellitus; HTN, hypertension.

* Patient groups sharing the same letter (a, b, or c) are not significantly (*P* > 0.05) different.

† Values were unknown for 24 BRDME patients and five BRAMD patients.

‡ HbA1c values were unknown for 51 BRDME patients.

§ Non-responders, <30% change; slow responders, 30%–70% change; high responders, >70% change.

### Variants Associated With Change in Visual Acuity

**Figure 1.**
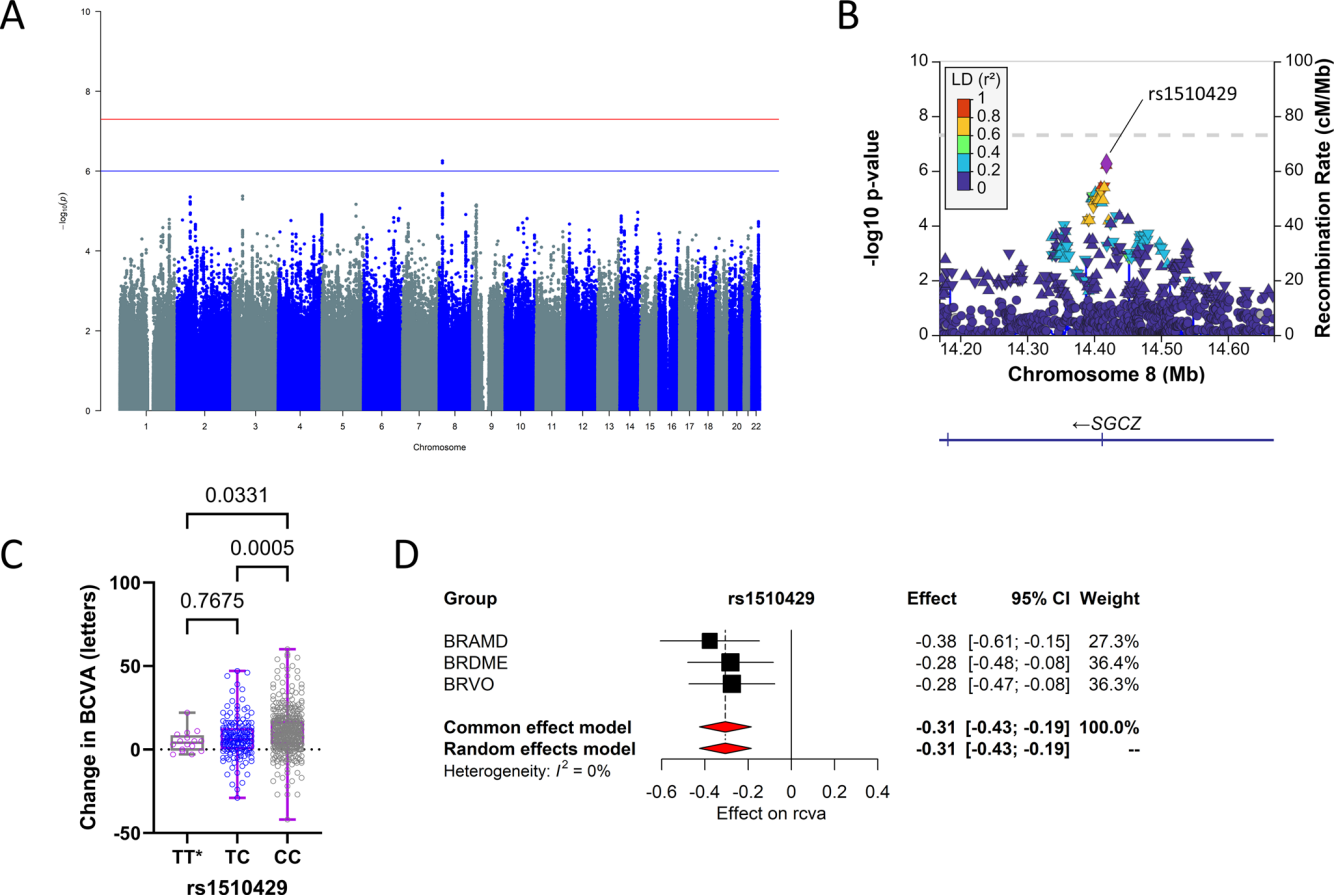
Variants associated with change in visual acuity. (**A**) Manhattan plot of GWAS for common variants in the combined cohort. The *blue line* indicates the suggested threshold (*P* < 1 × 10^−6^), and the *red line* indicates the genome-wide significance (*P* < 5 × 10^−8^). (**B**) Locus zoom plot of SNPs that are in the *SGCZ* gene on chromosome 8. (**C**) Box plots showing the effect on (rank-transformed) changes in BCVA after 6 months of treatment stratified by genotype. *P* values of Kruskal–Wallis tests are shown. TT* represents the least frequent genotype. (**D**) Forest plot for rs1510429 showing the effect on changes in BCVA per effect allele (T) in the three patient groups.

GWAS meta-analysis for the change in visual acuity after 6 months of anti-VEGF treatment in the combined patient groups resulted in a single genomic locus with *P* < 1 × 10^−^^6^ (Fig. 1). The corresponding quantile–quantile (Q–Q) plot is provided in Supplementary Figure S2A. The lead SNP, rs1510429, was found within the locus of the sarcoglycan zeta (*SGCZ*) gene on chromosome 8p22 (Table 2; Fig. 1B). A beneficial effect was observed for the most common CC genotype (71%), resulting in a significantly greater positive change in BCVA compared to the less frequent TT (3%) and TC (26%) genotypes (Fig. 1C). This variant was associated with a decrease in BCVA at 6 months of anti-VEGF treatment in all three patient groups (Fig. 1D).

**Table 2.** Most Significant Single Variants (*P* < 1 × 10^−^^6^) Associated With Change in BCVA or CST

Genomic Locus	SNP^*^	chr:pos	EA	NA	EAF	EA/EA	EA/NA	NA/NA	*P*	Dir	Nearest Gene	CADD
Change in BCVA												
1	rs1510429	8:14418703	T	C	0.16	4 (0, 8)	6 (0, 12)	10 (3, 17)	5.50E−07	− − −	*SGCZ*	0.644
Change in CST												
1	rs13145439	4:126103475	C	T	0.16	−113 (−206, −30)	−117 (−247, −45)	−155 (−283, −70)	8.19E−08	+ + +	*FAT4*	0.325
1	rs80174004	4:126125674	G	T	0.06	−87 (−136, −36)	−108 (−206, −44)	−145 (−282, −67)	1.85E−07	+ + +	*FAT4*	3.108
2	rs4872233	8:24283474	A	C	0.12	−46 (−148, 11)	−138 (−295, −65)	−139 (−272, −62)	1.20E−08	+ + +	*ADAMDEC1* and *ADAM7*	5.012
2	rs10448073	8:24288059	C	T	0.19	−44 (−188, 10)	−157 (−309, −80)	−135 (−262, −61)	1.10E−07	+ + +	*ADAMDEC1* and *ADAM7*	5.570
3	rs74915394	8:49618248	T	C	0.10	−176 (−251, 133)	−122 (−272, −31)	−142 (−276, −68)	1.35E−08	+ + +	*EFCAB1*	0.270
4	rs55674412	9:133406966	T	A	0.06	−89 (−225, 54)	−105 (−201, −22)	−146 (−284, −65)	1.07E−07	+ + +	*ASS1*	3.308
5	rs2279755	10:127942266	C	G	0.27	−121 (−249, −49)	−134 (−279, −46)	−151 (−277, −76)	1.96E−07	+ + +	*ADAM12*	0.328
6	rs3914794	18:2441133	A	G	0.06	−424 (−439, −409)	−66 (−223, −34)	−143 (−282, −69)	6.76E−07	+ + +	*METTL4*	3.006
Relative Change in CST												
1	rs12041929	1:63819985	A	T	0.43	47.0 (21.7, 74.5)	60.9 (28.0, 81.4)	65.2 (42.0, 82.6)	7.66E−07	− − −	*ALG6*, *FOXD3*	0.972
2	rs1643540	5:110839250	T	A	0.26	73.4 (51.1, 84.8)	62.2 (37.8, 81.5)	57.2 (27.4, 80.4)	8.26E−07	+ + +	*STARD4*, *CAMK4*	4.962
3	rs16908737	8:139285073	A	G	0.30	41.5 (16.3, 71.5)	56.0 (27.4, 76.9)	68.3 (42.5, 84.5)	4.46E−07	− − −	*FAM135B*	3.360
4	rs7239132	18:72226809	A	C	0.38	54.6 (16.1, 76.2)	57.3 (27.7, 79.6)	65.2 (42.4, 83.2)	3.80E−08	− − −	*CNDP1*	1.489

chr:pos, chromosome:position (GRCh37/hg19); Dir, direction: BRDME, BRVO, BRAMD; CADD, combined annotation dependent depletion. *P* < 5.0E-08 are genome-wide significant and indicated in bold. EA, effect allele; EAF, effect allele frequency; NA, non-effect allele.

* Minimal association factor (MAF) ≥ 5%. Change in BCVA = ETDRS letters/allele; change in CST = µm/allele; relative change in CST = change in CST as compared to baseline in percentages. Values for EA/EA, EA/NA, and NA/NA are presented as medians (Q1, Q3) per genotype.

### Variants Associated With Absolute Change in Retinal Thickness

**Figure 2.**
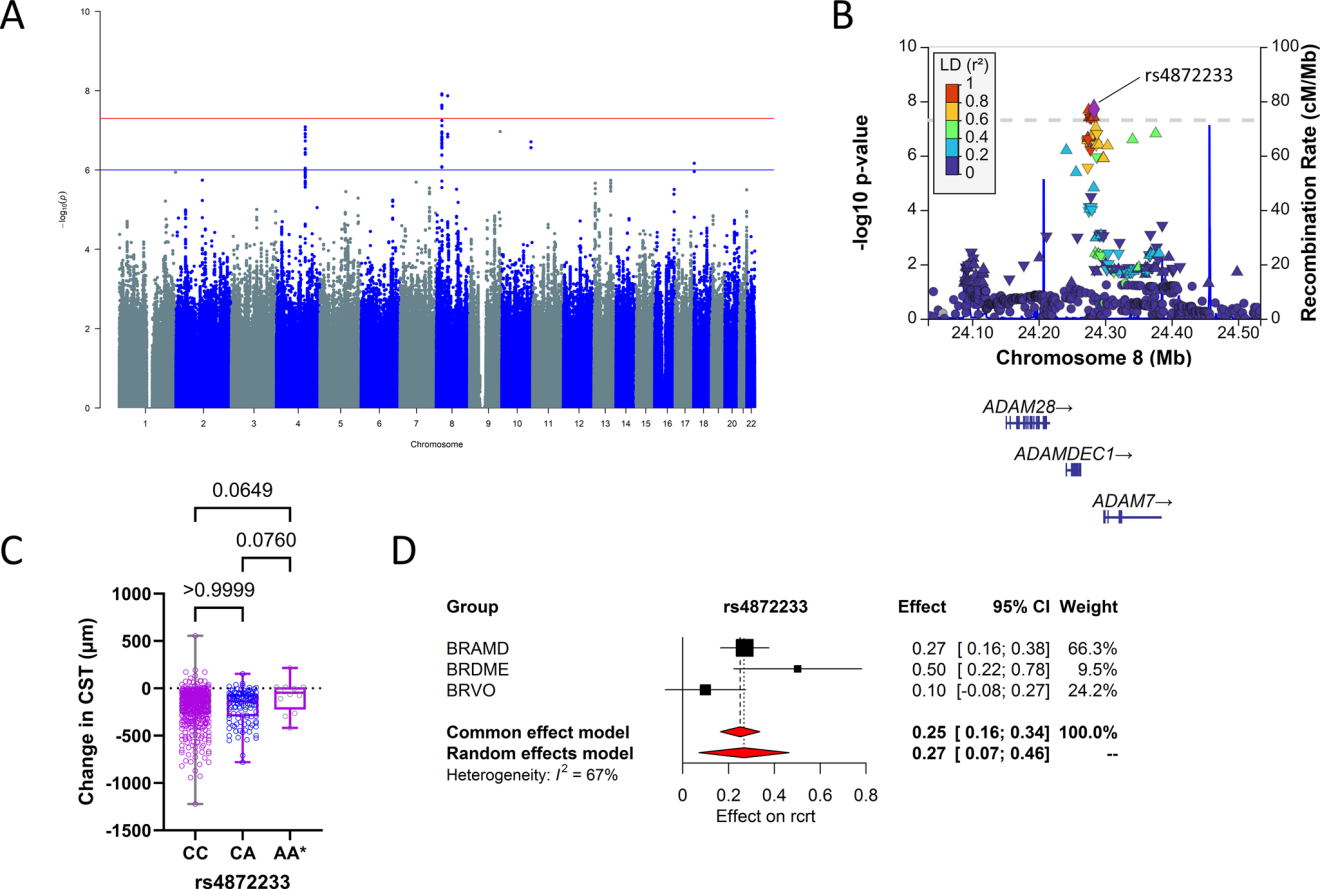
Variants associated with absolute change in CST. (**A**) Manhattan plot of GWAS for common variants the combined cohort. The *blue line* indicates the suggestive threshold (*P* < 1 × 10^−6^), and the *red line* indicates the genome-wide significance (*P* < 5 × 10^−8^). (**B**) Locus zoom plot of SNPs near the *ADAM7* and *ADAMDEC1* genes on chromosome 8. (**C**) Box plots showing the effect of (rank-transformed) change in CST after 6 months of treatment stratified by genotype. *P* values of Kruskal–Wallis tests are shown. AA* represents the least frequent genotype. (**D**) Forest plot for rs4872233 showing the effect on changes in CST per effect allele (T) in the three patient groups.

For changes in retinal thickness, we first looked at the absolute difference (in µm) of CST values at 6 months as compared to baseline. GWAS meta-analysis for change in retinal thickness after 6 months of anti-VEGF treatment for the combined patient groups resulted in six genomic loci with eight independent SNPs with *P* < 1 × 10^−^^6^ (Table 2; Fig. 2). The corresponding Q–Q plot is given in Supplementary Figure S2B. Two SNPs, located on chromosome 8, reached genome-wide significance (*P* < 5 × 10^−^^8^): rs4872233 and rs74915394. The variant on genomic locus 2 grouped closely together with another statistically significant SNP (rs10448073), near to the ADAM (a disintegrin and metalloproteinase) metallopeptidase domain 7 (*ADAM7*) and *ADAMDEC1* gene and also in the near vicinity of the *ADAM28* gene (Fig. 2B). The A-allele (effect allele frequency = 0.12) of SNP rs4872233 was related to an increase in CST at 6 months of anti-VEGF treatment in all three study groups (Figs. 2C, 2D). On chromosome 10, a variant in another ADAM gene (*ADAM12*) was identified (Table 2; Supplementary Fig. S3D). The other genome-wide significant lead SNP, rs74915394, was located on a second locus on chromosome 8, near to the EF-hand calcium binding domain 1 (*EFCAB1*) gene (Supplementary Fig. S3B). A locus on chromosome 4 with two SNPs (rs13145439 and rs80174004) near the FAT atypical cadherin 4 (*FAT4*) gene (Supplementary Fig. S3A), a SNP on chromosome 9 (rs55674412) near the argininosuccinate synthase 1 (*ASS1*) gene (Supplementary Fig. S3C), and a SNP on chromosome 18 (rs3914794) near the methyltransferase 4 (*METTL4*) gene (Supplementary Fig. S3E) reached statistical significance, as well.

### Variants Associated With Relative Change in Retinal Thickness

**Figure 3.**
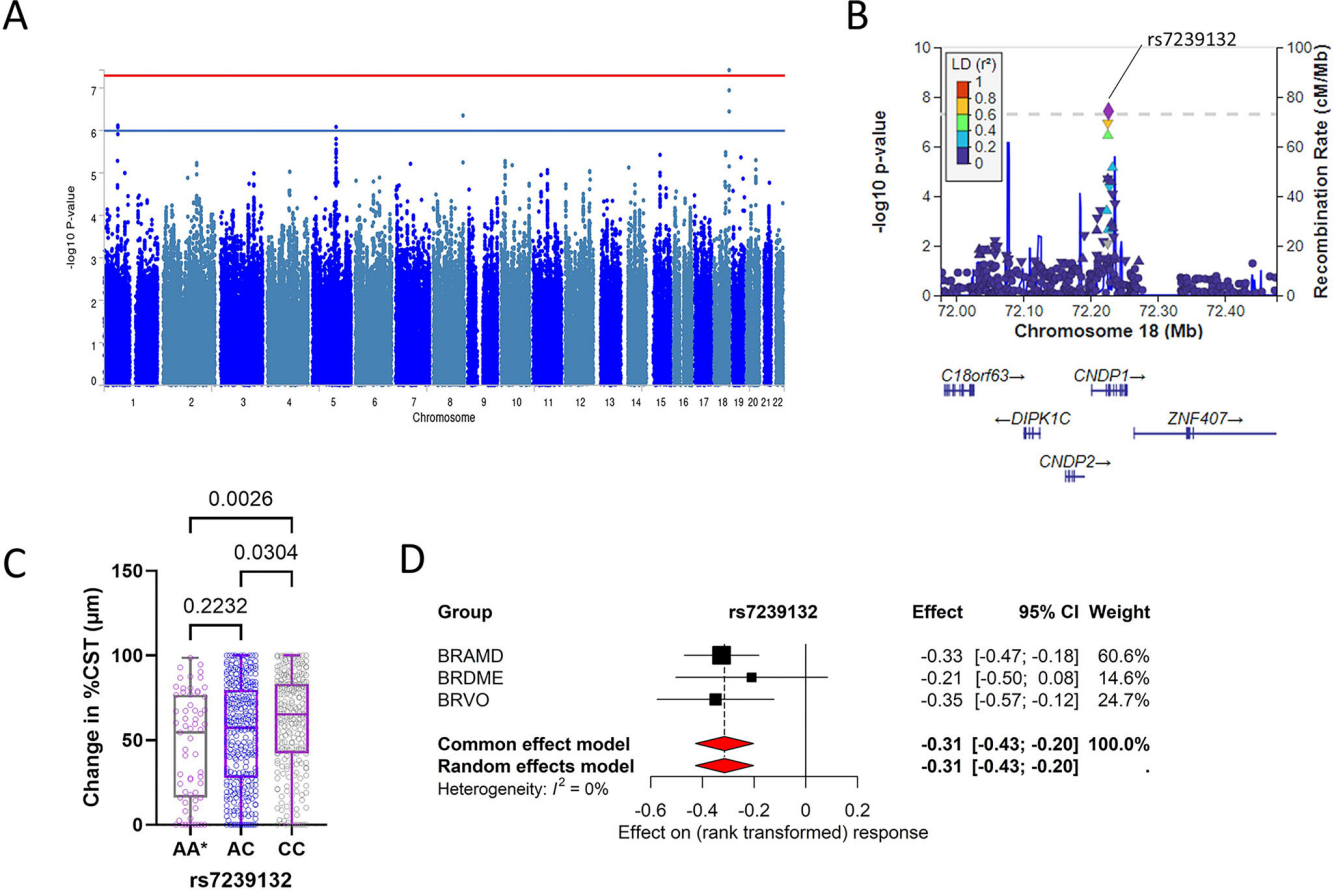
Variants associated with relative change in retinal thickness. (**A**) Manhattan plot of genome-wide association study for common variants the combined cohort. The blue line indicates the suggestive threshold (*P* < 1 × 10^−6^), the red line indicates the genome-wide significance (*P* < 5 × 10^−8^). (**B**) Locus zoom plot of single-nucleotide polymorphisms that are near the *CNDP1* gene on chromosome 18. (**C**) Box plots showing the effect of relative change in CST after 6 months of treatment stratified by genotype. *P* values of Kruskal-Wallis tests are shown. AA* represents the least frequent genotype. (**D**) Forest plot for rs7239132 showing effect on change in CST per effect allele (**A**) in the three patient groups.

To calculate the relative change in retinal thickness, we first established a threshold CST value for each study group. After subtracting this threshold from the measured CST, we determined the percentage change in the corrected CST values at 6 months from the corrected CST values at baseline which were set at 100% (see Supplementary Materials for details). GWAS meta-analysis for the change in retinal thickness after 6 months of anti-VEGF treatment in the combined patient groups resulted in the identification of four genomic loci with four independent SNPs, all having *P* < 9 × 10^−^^7^ (Table 2; Fig. 3). The corresponding Q–Q plot is provided in Supplementary Figure S2C. One genome-wide significant locus was represented by a SNP on chromosome 18 (rs7239132). This variant was found in the locus of the carnosine dipeptidase 1 (*CNDP1*) gene (Fig. 3B). Other SNPs were found on chromosomes 1, 5, and 8 (Table 2; Supplementary Fig. S4).

### Gene Expression in Retinal Cells and Tissues

**Figure 4.**
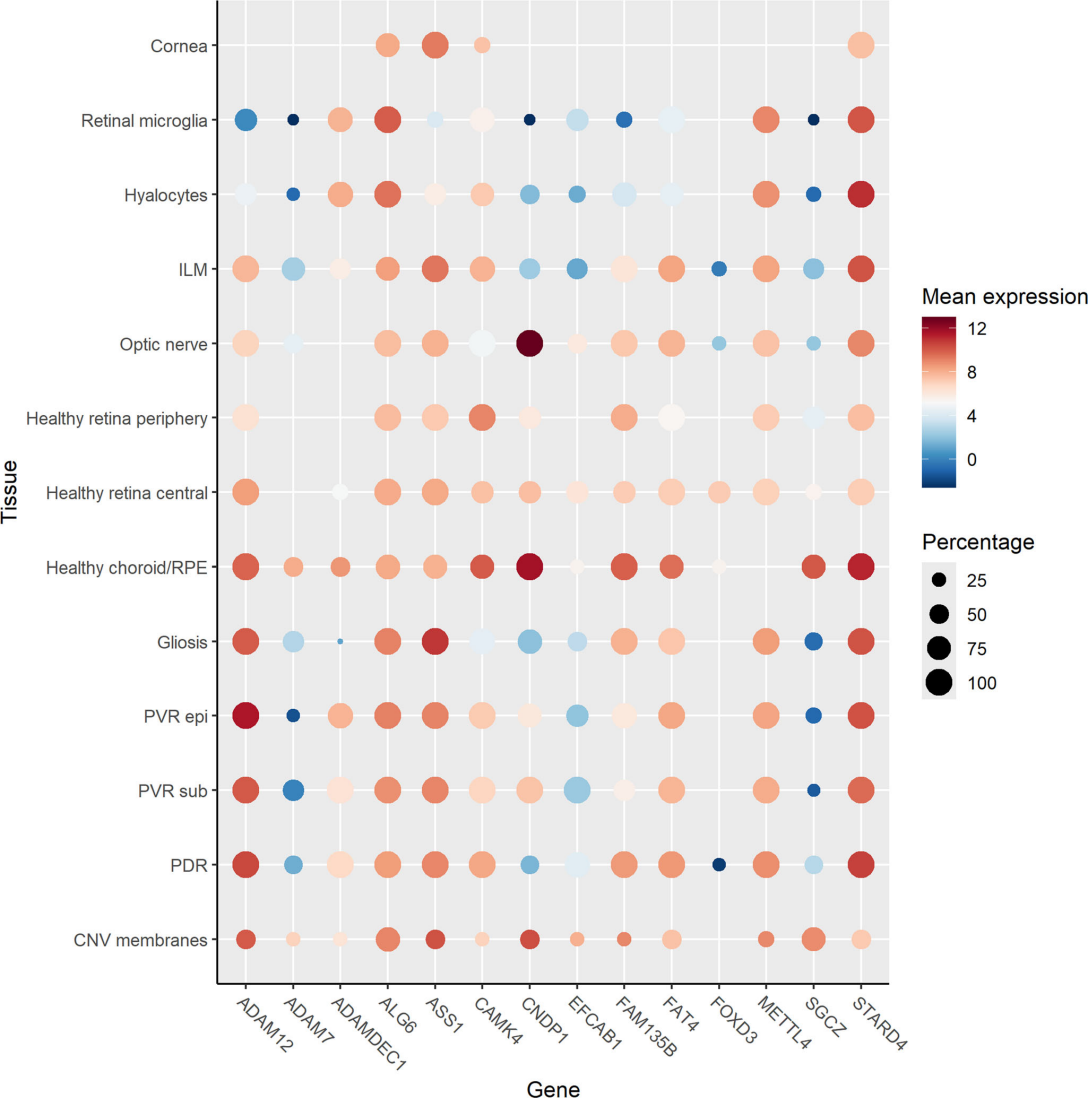
Dot plot of physiological and pathological gene expression in retinal cells and tissue. Data were extracted from single-cell RNA sequencing data of the Human Eye Transcriptome Atlas.^26^ Normalized RNA expression levels (nTPM) are shown for eight healthy retinal cells and tissues and in five retinal disease states. *Color coding* is based on expression levels in log_2_ scale, and the *size of* *the* *dots* is based on the percentage of samples with detectable expression levels.

Several candidate genes were identified. To determine their expression patterns in ocular tissues or specific cells, we referred to the Human Eye Transcriptome Atlas,^26^ and the results are summarized in Figure 4. All genes showed expression in healthy choroid/retinal pigment epithelium (RPE) and almost all, except *ADAM7*, in healthy central retina. Forkhead box D3 (*FOXD3*) transcription factor expression was not found in pathological conditions.

### Previously Reported SNPs and Targeted Gene Approach

We then focused on genetic variants and regions previously reported to be associated with anti-VEGF response (Table 3). We observed a significant positive association between changes in BCVA and variants of the VEGFR2 (*KDR*), interleukin 6 (*IL6*), and neuropillin 1 (*NRP1*) genes in all patient groups, but no associations were found with changes in CST for these genes. For the sphingosine-1-phosphate lysolipid transporter 2 (*SPNS2*) gene variant, we observed a positive association with changes in BCVA in the BRDME and BRAMD groups but a negative association in the BRVO group. Additionally, we concentrated on several genes presumed to play a role in the pathophysiology of macular edema: *VEGFA*, *VEGFC*, *EPO*, *NOS3* (eNOS), *PLVAP*, and *APOE4* (Table 4). Among these genes, a SNP in *PLVAP* demonstrated a positive association with absolute changes in CST for rs73016427 in all three patient groups. No associations with SNPs in the other genes were observed.

**Table 3.** Gene Polymorphisms Previously Associated With Anti-VEGF Treatment Response

							CVA		CST		PCST			
Gene	SNP	Chr:Position	REF	ALT	AAF	EA	*P*	Dir	*P*	Dir	*P*	Dir	Association	Study
*CCT3*	rs12138564	1:156291600	G	A/T	0.163	T	0.647	+ − −	0.668	− + −	0.478	+ − −	Anti-VEGF response BCVA in nAMD	Lorés-Motta et al.^4^
*HPCAL1*	rs11888704	2:10530325	G	T	0.084	T	0.417	+ − −	0.083	+ + +	0.048	− − −	Anti-VEGF response CST in DME	Hong et al.^14^
*DIRC3*	rs16857280	2:218175073	G	A	0.078	A	0.994	+ + −	0.558	+ − +	0.503	− + +		
*VEGFR2*	rs6828477	4:55966801	C	T	0.588	T	**0.003**	+ + +	0.559	+ − −	0.756	− + −	Anti-VEGF response VA in nAMD	Hermann et al.^15^
*PGAM1P1*	rs18786551	5:57535905	G	A	0.010	A	NA	NA	NA	NA	NA	NA	Anti-VEGF response VA in DME	Gurung et al.^8^
*COL23A1*	rs17081072	5:177747691	T	C	0.131*	C	0.941	+ + −	0.558	− − −	0.690	− − +	Anti-VEGF response CST in DME	Hong et al.^14^
*CASC15*	rs78466540	6:21755718	A	G	0.025	G	NA	NA	NA	NA	NA	NA	Anti-VEGF response CMT in DME	Gurung et al.^8^
*SLC35F1*	rs11153718	6:118372057	G	A	0.240	A	0.976	− − +	0.416	− + −	0.107	+ + +	Anti-VEGF response CST in DME	Hong et al.^14^
*TBC1D32*	rs118074968	6:121280000	A	G	0.021	G	NA	NA	NA	NA	NA	NA	Anti-VEGF response BCVA in DME	Gurung et al.^8^
*IL6*	rs2069845	7:22770149	G	A/C/T	0.539	G	**0.043**	+ + +	0.325	− − −	0.271	− − +	Anti-VEGF response BCVA in nAMD	Burés Jelstrup et al.^16^
*IGF2BP3*	rs12700428	7:23436427	G	A	0.272	A	0.673	− + +	0.199	+ + +	0.901	− + +	Anti-VEGF response CST in DME	Hong et al.^14^
*CREB5*	rs4722804	7:28532464	G	T	0.173	T	0.911	− − +	0.593	− + −	0.814	+ − −		
*RAB2A*	rs2272620	8:61452046	A	G	0.393*	A	0.895	− + −	0.266	− + −	0.689	+ − +		
*KIAA0368*	rs10759497	9:114220603	C	T	0.618	T	0.347	− + +	0.492	− − −	0.051	+ + +		
*NRP1*	rs2070296	10:33552695	C	C	0.162	C	**0.039**	+ + +	0.994	+ + −	0.452	+ + −	Anti-VEGF response BCVA in nAMD	Lorés-Motta et al.^17^
*C10orf88*	rs115539014	10:124692082	C	T	0.000	RV	NA	NA	NA	NA	NA	NA	Anti-VEGF response BCVA in nAMD	Lorés-Motta et al.^4^
* *	rs371923396	10:124712511	C	T	0.000	RV	NA	NA	NA	NA	NA	NA		
*OR52B4*	rs4910623	11:4389639	G	A/T	0.486	A	0.066	+ + +	0.756	− − +	0.866	+ + −	Anti-VEGF response BCVA in nAMD	Riaz et al.^18^
*UNC93B1*	rs112921257	11:67765163	A	G/T	1.000	RV	NA	NA	NA	NA	NA	NA	Anti-VEGF response BCVA in nAMD	Lorés-Motta et al.^4^
	rs146593182	11:67770499	G	T	0.003	RV	NA	NA	NA	NA	NA	NA		
*RP11-116D17.1*	rs11615833	12:115772032	G	A	0.077	A	0.107	+ − −	0.652	− + +	0.792	+ − +	Anti-VEGF response CMT in DME	Gurung et al.^8^
	rs11614480	12:115772072	T	C	0.094	C	0.107	+ − −	0.652	− + +	0.793	− + −		
	rs11615848	12:115772088	G	T	0.090	T	0.107	+ − −	0.652	− + +	0.793	+ − +		
	rs11615870	12:115772214	G	T	0.056	T	0.107	+ − −	0.652	− + +	0.793	+ − +		
	rs11614887	12:115772313	T	C	0.098	C	0.107	+ − −	0.652	− + +	0.792	− + −		
*TMEM132D*	rs155370	12:130077096	T	C	0.376	C	0.928	+ − −	0.962	− − +	0.080	− − −	Anti-VEGF response CST in DME	Hong et al.^14^
*SLCO3A1*	rs12899055	15:92604530	T	C	0.659	C	0.927	− − +	0.511	+ + −	0.108	+ + −		
*SPNS2*	rs7213707	17:4414027	A	G	0.776*	G	**0.023**	+ − +	0.388	− + −	0.680	− − −		

ALT, alternative allele; AAF, alternative allele frequency in Europeans; CVA, change in visual acuity (BCVA); Dir, direction; CST, change in central subfield thickness in absolute values; EA, effect allele; NA, not applicable (*P* values are not applicable or not available due to calculation difficulties); PCST, percent change in central subfield thickness; RV, rare variant; Significant *P* values (<0.05) are indicated in bold. REF, reference allele

**Table 4.** Gene Polymorphisms Previously Associated With DR With Presumed Function in Macular Edema

							CVA		CST		PCST			
Gene	SNP	Chr:Position	REF	ALT	AAF	EA	*P*	Dir	*P*	Dir	*P*	Dir	Association	Study
*VEGFC*	rs17697419	4:177608166	G	A	0.107	A	0.828	− + −	0.740	− + +	0.455	+ − −	DME and DR	Kaidonis et al.^19^
*VEGFA*	rs699946	6:43732669	A	G	0.196	A	0.451	+ + +	0.946	+ − −	0.814	− + +	DR and CSME	Abhary et al.^20^
	rs2010963	6:43738350	C	G/T	0.670	C/G	0.229	+ − −	0.862	− − +	0.483	+ − +	DR	Tetikoğlu et al.^21^
	rs833068	6:43742527	G	A	0.329	G	0.288	− + +	0.883	+ + −	0.408	+ − +	DR	Abhary et al.^20^
	rs10434	6:43753212	A	G/C/T	0.540	C/G	0.956	− + +	0.539	+ + −	0.800	+ + +	DR	Tetikoğlu et al.^21^
*EPO*	rs1617640	7:100317298	C	A/G/T	0.534	G	0.950	+ − +	0.679	− + +	0.459	− + −	DR	Abhary et al.^22^
	rs507392	7:100319936	G	A/C	0.582	C	0.969	+ − +	0.701	− + +	0.452	− + −		
	rs551238	7:100321528	G	C/T	0.582	C	0.969	+ − +	0.701	− + +	0.452	− + −		
*eNOS*	rs2070744	7:150690079	C	G/T	0.627	C	0.173	− − −	0.063	+ + −	0.951	+ + −	DME	Awata et al.^23^
*PLVAP*	rs73016427	19:17468090	G	A	0.106	A	0.819	− + −	**0.034**	+ + + ++	0.907	− + −	DR	Stockwell et al.^24^
*ApoE4*	rs429358	19:45411941	T	C	0.068	E4 (C)	0.901	− − +	0.573	− + −	0.483	− + +	DR	Dlouha et al.^25^
	rs7412	19:45412079	C	T	0.083	E2 (T)	0.298	− + −	0.877	+ − −	0.985	− + +		

AAF, alternative allele frequency in Europeans; ALT, alternative allele; CSME, clinically significant macular edema; CST, change in central subfield thickness in absolute values; CVA, change in visual acuity (BCVA); Dir, direction; EA, effect allele; PCST, percent change in central subfield thickness; REF, reference allele.

Significant *P* values (<0.05) are indicated in bold.

## Discussion

In this study, we identified multiple loci associated with functional and anatomical anti-VEGF response in 606 patients with macular edema, encompassing three distinct retinal conditions with varying primary pathologies. Patients with DME, RVO, or nAMD share the common feature of macular edema, a significant cause of visual impairment and a primary target of anti-VEGF therapy. Given the variability in treatment response among these patients, we hypothesized that specific genetic variations common to all conditions may contribute to these differences in outcomes. Our study design specifically focused on outcomes related to the shared pathology of VEGF-driven macular edema, thus mitigating limitations associated with smaller sample sizes. We correlated the results of a GWAS meta-analysis with changes in BCVA and CST 6 months after initiating monthly anti-VEGF therapy. By combining the three patient groups, we enhanced the statistical power of our analysis and were able to compare effects across patient groups, where homogeneity or lack of heterogeneity could be regarded as an internal validation.

Our GWAS meta-analysis revealed that rs1510429-T in the *SGCZ* gene on chromosome 8p is negatively correlated with changes in BCVA following anti-VEGF treatment. Carriers of the T-allele are more likely to be non-responders based on the change in BCVA. SGCZ is a component of the sarcoglycan complex, which consists of six proteins forming the dystrophin-associated glycoprotein complex (DGC). This complex connects the inner cytoskeleton to the extracellular matrix. *SGCZ* has previously been associated with body mass index,^27^ obesity-related traits in African Americans,^28^ and prediabetes status change.^29^ However, further investigation is needed to understand how SGCZ may be related to the response to anti-VEGF therapy in retinal conditions with macular edema, particular as BCVA loss is a functional indirect effect of VEGF-induced ME and choroidal neovascularization.

For the change in CST, we employed two distinct strategies of using either the absolute change in microns or the relative change in percentages. Given that baseline CST values are relatively higher in some patients, we anticipated that each strategy might reveal different genetic associations. This expectation was confirmed in our findings. In our meta-analysis, an association of absolute change in CST was found with genes of the ADAM gene family. *ADAM7* and *ADAMDEC1* are positioned on the same locus at chromosome 8, also in the vicinity of *ADAM28*, and *ADAM12* was mapped on chromosome 10. The ADAM gene family is part of the zinc metalloproteases, which also include matrix metalloproteinases (MMPs) and a disintegrin and metalloproteinase with thrombospondin motifs (ADAMTSs).^30^ They play important roles in developmental processes and cell–matrix interactions. Typical substrates of ADAM proteases are cytokines, chemokines, growth factors, and their receptors, as well as cell adhesion molecules and differentiation factors.^31^ ADAM12 was previously found to be essential in hypoxia-induced breakdown of the neurovascular barrier in brain microvascular endothelial cells and mouse retina^32^ and is upregulated in ischemia.^33^ In vitro and in vivo, it was demonstrated that ADAM12 mediates shedding of Tie2 and to a lesser extent that of vascular endothelial (VE)-cadherin, and VEGFR2.^33^ All of these findings together provide clear evidence for the probable involvement of the ADAM gene family in the development of macular edema, due to their role in BRB breakdown and hypoxia and VEGF-related processes. Also noteworthy to mention is that *ASS1* regulates the delivery of l-arginine, the substrate of eNOS.^34^ Mutations in *FAT4* have been associated with Van Maldergem syndrome and Hennekam syndrome, rare diseases with lymphedema.^35^ *FAT4* is important for lymphatic vessel morphogenesis during development, as the majority of homozygous FAT4-deficient embryos exhibited subcutaneous edema.^36^

An association with relative changes in CST in our meta-analysis was found on three gene loci: *CNDP1*, *STARD4*, and *FAM135B* and in the vicinity of three other genes: *ALG6* (alpha-1,3-glucosyltransferase), *FOXD3*, and *CAMK4* (calcium/calmodulin-dependent protein kinase IV). Carnosine, which is regulated by CNDP1, is an abundant antioxidant in the retina and present in retinal neurons. Oral carnosine supplementation has shown protective effects against vascular damage in diabetic retinopathy in animal studies.^37^ STARD4 (STAR-related lipid transfer domain protein 4) plays a significant role in cholesterol metabolism and transfer within the retina.^38^ Not much is known about *FAM135B* (family with sequence similarity 135 member B), but according to National Center for Biotechnology Information (NCBI) Gene Cards, it is predicted to play a role in lipid metabolism (provided by Alliance of Genome Resources, April 2022). *ALG6* encodes alpha-1,3-glucosyltransferase, an enzyme that is involved in protein *N*-glycosylation. Mutations in this gene have been associated with retinitis pigmentosa type 59 (RP59).^39^ RP59 is primarily caused by a missense mutation in *DHDDS* (encoding dehydrodolichyl diphosphate synthase), an enzyme essential for *N*-glycosylation across all cell types. *ALG6* has been identified as a genetic modifier in RP59, with sequence variations correlating with phenotypic variability among patients. However, the SNP identified in our study (*rs12041929*) lies near *ALG6* and therefore does not represent this specific functional variant. FOXD3 plays an important role in neural crest development, which contributes to anterior eye structures. This suggests that FOXD3 could potentially be involved in eye development and disease.^40^ Finally, CAMK4, also known as CaMKIV or Ca^2+^/calmodulin-dependent protein kinase IV, plays a significant role in retinal function and has been implicated in various processes within the retina. Its activity is linked to the regulation of important substrates like CREB (cAMP response element-binding protein) and is reduced in retinal degenerative conditions, suggesting its involvement in maintaining photoreceptor health.^41^ Furthermore, CAMK4 was found to play a pivotal role in blood pressure regulation through the control of eNOS activity.^42^ Further research is needed to understand the respective roles of these molecules in macular edema and anti-VEGF response.

In addition to a discovery-based GWAS analysis, we also employed a more targeted approach by looking at individual SNPs and SNPs in genes previously associated with our outcomes. For this purpose, we delved into recent literature to identify SNPs from genes associated with anti-VEGF response, or with known or presumed functions in the molecular pathways underlying the pathophysiology of ME. Several earlier studies have explored genetic variants in relation to anti-VEGF response. Among these studies, the work of Lorés-Motta et al.^4^ stands out as the largest, encompassing 2058 patients. However, we encountered difficulties in replicating their results. It is worth noting that our study identified a higher number of SNPs associated with CST change in relation to anti-VEGF treatment response, whereas the study by Lorés-Motta et al.^4^ focused solely on BCVA change in neovascular AMD, which is likely to be influenced by many other factors than VEGF-driven ME, including subretinal neovascularization, RPE atrophy, and wound healing processes such as fibrosis.^43^ The other studies may have been underpowered due to a low number of participants,^44^ and/or variants may have been missed by a different ethnical minimal allele frequency.^8^^,^^14^

In our targeted approach, we were able to identify gene variants in four genes that displayed a significant association with changes in BCVA: VEGFR2 (*KDR*), *IL6*, *NRP1*, and *SPNS2*, as well as one gene associated with changes in CST: *PLVAP*. Both VEGFR2 and NRP1 serve as receptors for VEGF, strongly supporting a significant relationship between variants in these genes and the regulation of anti-VEGF responsiveness.^15^^,^^17^ IL6 plays a crucial role in retinal inflammation and macular edema by inducing VEGF production and facilitating vascular leakage through the downregulation of tight junction proteins in retinal endothelial cells.^16^^,^^45^ SPNS2 is involved in transporting sphingosine 1-phosphate (S1P) and has been linked to impaired blood–brain barrier function in ischemia–reperfusion injury.^46^ S1P has recently emerged as a potential target in neovascular retinal diseases.^47^ PLVAP, which is normally absent under stable BRB conditions, appears in retinal endothelial cells under pathological conditions, especially in conditions with edema.^48^ The association of an anatomical anti-VEGF response with *PLVAP* is not unexpected, given its upregulation in endothelial cells induced by VEGF and hypoxia, both in vitro and in vivo, and its direct role in VEGF-induced barrier loss.^49^^,^^50^ This makes PLVAP a potential therapeutic target.^50^ It is important to note that the association of these genes loses statistical significance after Bonferroni correction. However, these findings align with the hypothetically expected association with anti-VEGF response in ME.

The limitations of our study include a relatively small sample size, which may result in limited statistical power (see Power Statement) and an increased likelihood of false-negative results. Additionally, we were unable to validate our findings in an external reference cohort due to the limited availability of published studies on this topic. However, the strength of our study lies in the combination of three groups of patients with different retinal vascular diseases—DME, RVO, and nAMD—all of which share macular edema as a common feature against a background of different primary pathologies and which can also serve as independent reference cohorts amongst each other. Our hypothesis was that these three diseases share similarities in the development of ME and also exhibit a similar response to anti-VEGF therapy.

## Conclusions

We identified one SNP on one locus within the *SGCZ* gene that was associated with changes in BCVA, eight SNPs on six loci associated with absolute changes in CST, and four SNPs on four loci associated with relative changes in CST after 6 months of anti-VEGF treatment. One locus associated with absolute change in CST was in the *ADAM12* gene, which is highly related to biological processes in vascular permeability. Remarkably, associations were also found with different loci in close proximity to other ADAM genes, *ADAM7*, *ADAM28*, and *ADAMDEC1*. Four SNPs on four loci associated with relative changes in CST were associated with metabolic processes, including glycosylation and cholesterol metabolism and relevant roles in retinal functions or eNOS-related vascular permeability. Furthermore, through targeted GWAS analysis, we identified significant associations between changes in BCVA and variants in four genes: *VEGFR2* (*KDR*), *NRP1*, *IL6*, and *SPNS2*. Additionally, we found an association between changes in CST and the *PLVAP* gene. All of these genes are known to be crucial in VEGF signaling and vascular permeability regulation. Although these identified loci require further validation, they hold the potential to serve as predictors of treatment outcomes for anti-VEGF therapy in ME and provide further insight in the pathogenesis of VEGF-driven ME by the potential identification of unknown pathways.

## Supplementary Material

Supplement 1
