# Supplementary material for: Several Common Genetic Variations Associate With Functional or Anatomic Effects of Anti-VEGF Treatment in Conditions With Macular Edema
Source: Invest Ophthalmol Vis Sci. 2025 Jun 2;66(6):2. doi: 10.1167/iovs.66.6.2 (PMC12136111; doi:10.1167/iovs.66.6.2)
Supplement: Supplement 1 [file iovs-66-6-2_s001.pdf]

Supplementary material

Supplementary material and methods

Patient characteristics

Between January 2009 and December 2011, 327 patients were enrolled in the BRAMD study, including patients over 60 years of age with primary or recurrent sub- or juxtafoveal choroidal neovascularization (CNV) secondary to AMD with a total area of CNV of < 12 disc areas and a BCVA between 20 and 78 letters.[1] Patients received 12 monthly injections of either 1.25 mg bevacizumab (n = 161) or 0.5 mg ranibizumab (n = 166). Between June 2012 and February 2018, 170 participants were enrolled in the BRDME study, including patients with DME.[2] Patients were eligible for participation if over 18 years of age, diagnosed with type 1 or type 2 diabetes mellitus with a glycosylated hemoglobin (HbA1c) of less than 12%, a CST of >325 µm, and a BCVA between 24 and 79 letters. Patients received 6 monthly injections of either 1.25 mg bevacizumab (n = 86) or 0.5 mg ranibizumab (n = 84). In the same period from 2012 to 2018 a total of 286 patients were included in the BRVO study, including patients with vision loss resulting from ME secondary to a branch or (hemi) central RVO.[3] Other inclusion criteria were age older than 18 years, a CST >325 µm on OCT, and a BCVA between 24 and 79 letters. Patients received monthly injections of either 1.25 mg bevacizumab (n = 144) or 0.5 mg ranibizumab (n = 142). Follow-up time was 12 months in the BRAMD study and 6 months in the BRDME and BRVO studies.

Processing of blood samples

Whole blood samples were collected in PAXgene Blood DNA Tubes (Qiagen, Hilden, Germany) and DNA was automatically processed. The concentration and quality of DNA samples was assessed by photospectral quantification (Nanodrop) and visual inspection on agarose gels, respectively. DNA was aliquoted in samples of 200 ng in a maximum of 10 µl on 96-well plates.

Genotyping, imputation and quality controls

The DNA samples were genotyped using the Infinium® Global Screening Array (version 3), containing more than 700,000 single nucleotide polymorphisms (SNPs). This Illumina array has a very rich up-to-date content including rare variants, while also containing clinically relevant content and is therefore suitable for genome-wide association analysis (GWAS). The array was run at the Human Genomics

Facility (HuGe-F) at Erasmus Medical Center. Pre-imputation quality control was performed using PLINK (v2.0). SNPs were excluded if ambiguous (A/T or C/G), had missingness rates >0.05, Hardy-Weinberg equilibrium test P $<10^{-10}$ or minor allele frequency (MAF) <0.01. Samples with missingness >0.02, inbreeding coefficient F ≥0.1, as well as those with unresolved genotype-phenotype sex mismatch were excluded. Identity by descent (IBD) analysis was performed using the "—genome" function in PLINK. Samples with excessive rates of proportional IBD (PI-HAT) >0.05 were excluded. Principal component analysis was performed to adjust for the confounding factors of ancestry and ethnicity differences. Non-European ancestry samples were excluded based on inspection of scatter plots of the first 4 principal components (**Supplementary Fig S1**). After exclusion of the outliers, a second principal components analysis (PCA) was performed again using the remaining samples. Genome-wide imputation was performed using Eagle2 phasing, Minimac3 and the Haplotype Reference Consortium (HRCr1.1) panel implemented on the Michigan Imputation Server v.1.0.2. In the post imputation quality control, only SNPs with MAF >0.05 in each cohort and a Minimac R^2 >0.5 were taken forward in the association analysis (**Supplementary Table S1**).

Absolute and relative change in CST

Change in CST (in µm) was measured with Zeiss Cirrus (Carl Zeiss Meditec, Dublin, Ireland), Heidelberg Spectralis(Heidelberg Engineering, Heidelberg, Germany), or Topcon 3D (Topcon, Tokyo, Japan) spectral domain OCT. OCT values obtained by Zeiss Cirrus or Topcon devices were converted to Heidelberg Spectralis values for analysis and reporting, using the conversion table reported by Giani et al.[4].
We calculated the relative change in CST as a percentage from baseline to 6 months after subtracting an estimated minimal CST threshold from the CST values at both baseline and 6 months. The difference between the baseline CST and this threshold was set at 100%. Since the minimal CST varies among individuals and is unknown, using a fixed minimal CST of 283 µm, as in Vader et al. (2021), led to biased results. This fixed threshold caused negative baseline values in some cases for the BRAMD cohort and negative values after 6 months of anti-VEGF treatment for the BRVO cohort, as many patients had CST values below this threshold at 6 months. This misclassification turned non-responders into good responders.

To determine a valid subtraction value, we employed a step-down strategy to ensure an equal distribution of CST changes from 0% to 100%, ensuring that at least some patients achieved complete resolution of edema. For the BRDME and BRVO cohorts, the threshold was set at 225 µm, and for the

BRAMD cohort, it was set at 150 µm. The relative change in CST compared to the baseline was then used as an outcome measure, with and without cut-off points (values higher than baseline values set to 0% CST decrease and values on or below the threshold to 100% CST decrease).

Lead candidate genes

FUMA was used to conduct gene-based GWAS and gene-set pathway analysis. In the MAGMA gene-based GWAS, SNPs were mapped to 18533 protein-coding genes, and the resulting SNP *P* values were combined into a gene test statistic using the SNP-wise mean model. MAGMA gene set pathway analyses using the full distribution of SNP *P* values were performed for curated gene sets and Gene Ontology terms obtained from MSigDB (https://www.gsea-msigdb.org/gsea/msigdb/). Additionally, we included a number of genes that were in close genomic proximity based on LocusZoom plots. For the locus on chromosome 8 in relation to change in VA, only the *SGCZ* gene was found. In relation to absolute change of CST, four genes were identified by FUMA (*ADAM12*, *ADAM7*, *ADAMDEC1* and *EFCAB1*) and three additional genes by LocusZoom (*ASS1*, *FAT4* and *METTL4*). In relation to relative change of CST, four genes were identified by FUMA (*CAMK4*, *CNDP1*, *FAM135B*, *STARD4*) and two additional genes by LocusZoom (*ALG6* and *FOXD3*). To investigate the enrichment of genes in retinal tissues and cells, we queried the Human Eye Transcriptome Atlas (https://www.eye-transcriptome.com/index.php) (Wolf et al., 2022). From the Human Eye Transcriptome Atlas (accessed on July 13, 2024), we extracted nTPM values, calculated mean values, and determined the percentage of samples with detectable expression levels.

1. Schauwvlieghe AM, Dijkman G, Hooymans JM, et al. Comparing the Effectiveness of Bevacizumab to Ranibizumab in Patients with Exudative Age-Related Macular Degeneration. The BRAMD Study. *PLoS One* 2016;11:e0153052.
2. Vader MJC, Schauwvlieghe AME, Verbraak FD, et al. Comparing the Efficacy of Bevacizumab and Ranibizumab in Patients with Diabetic Macular Edema (BRDME): The BRDME Study, a Randomized Trial. *Ophthalmol Retina* 2020;4:777-788.
3. Vader MJC, Schauwvlieghe AME, Verbraak FD, et al. Comparing the Efficacy of Bevacizumab and Ranibizumab in Patients with Retinal Vein Occlusion: The Bevacizumab to Ranibizumab in Retinal Vein Occlusions (BRVO) study, a Randomized Trial. *Ophthalmol Retina* 2020;4:576-587.

4. Giani A, Cigada M, Choudhry N, et al. Reproducibility of retinal thickness measurements on normal and pathologic eyes by different optical coherence tomography instruments. *Am J Ophthalmol* 2010;150:815-824.

Table S1 Quality control (QC) steps in PLINK (1.9/2.0) and ADMIXTURE for individual array set.

Starting numbers	Post individual QC (PLINK)	Post-imputation QC (PLINK)
725830 SNPs<br>672 patients	511509 SNPs<br>606 patients<br><br>PLINK parameters:<br>--geno 0.02<br>(22989 SNPs removed)<br>--mind 0.05<br>(2 individuals removed)<br>--maf 0.01<br>(196329 SNPS removed)<br>--hwe 10^{-10}<br>(491 SNPs removed)<br>--sex<br>(0 individuals removed)<br>--het<br>(2 individuals removed)<br><br>Additional exclusions:<br>58 Non-European individuals<br>3 individuals with no phenotype<br>1 individual without macular oedema<br><br>5376 (A/T or C/G) SNPs removed	Common variants<br>5,145,653 SNPs remaining<br><br>Imputation parameters:<br>--mach-r2-filter 0.5<br>--maf 0.05 in each cohort<br><br>Additional exclusion:<br>P heterogeneity $< 10^{-7}$

SNP: Single nucleotide polymorphism; SNV: Single nucleotide variant

Figure S1

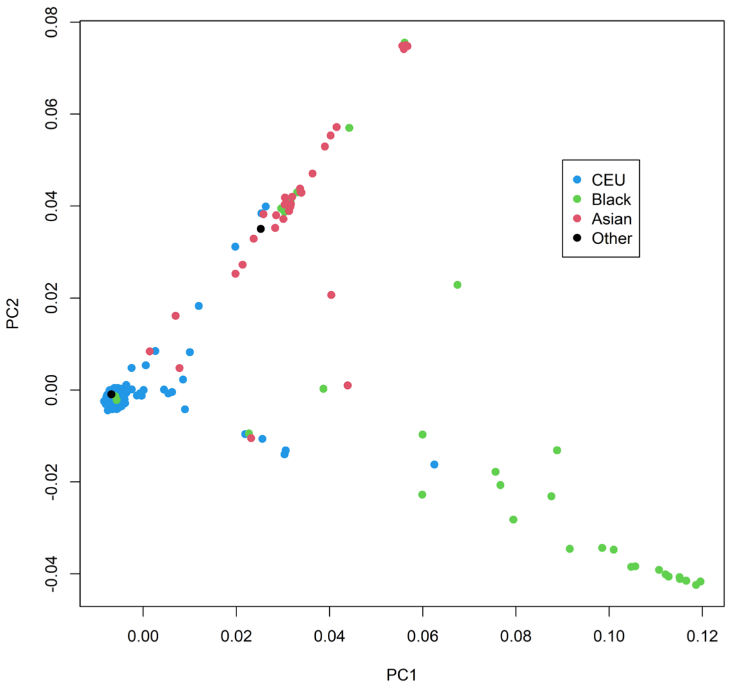

Figure S1. Principal component analysis to detect ethnic outliers. The x-axis represents the first principal component (PC1) and the y-axis is the principal component 2 (left). Colors correspond to self reported ethnicity. CEU includes Northwest Europeans.

Figure S2

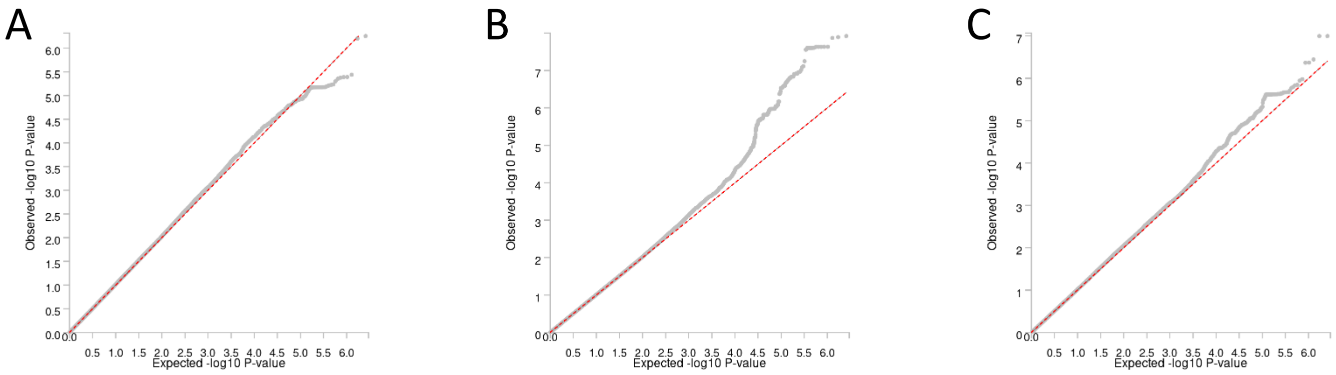

Figure S2. Quantile-Quantile plot for association analysis. (**A**) Change in best-corrected visual acuity. (**B**) Change in absolute central subfield thickness. (**C**) Change in relative central subfield thickness. The plots show the expected −log10 *P* values under the null hypothesis on the x-axis, and the observed −log10 *P* values on the y-axis. The red lines represent x=y, and the grey lines represent the 95% confidence interval.

Figure S3

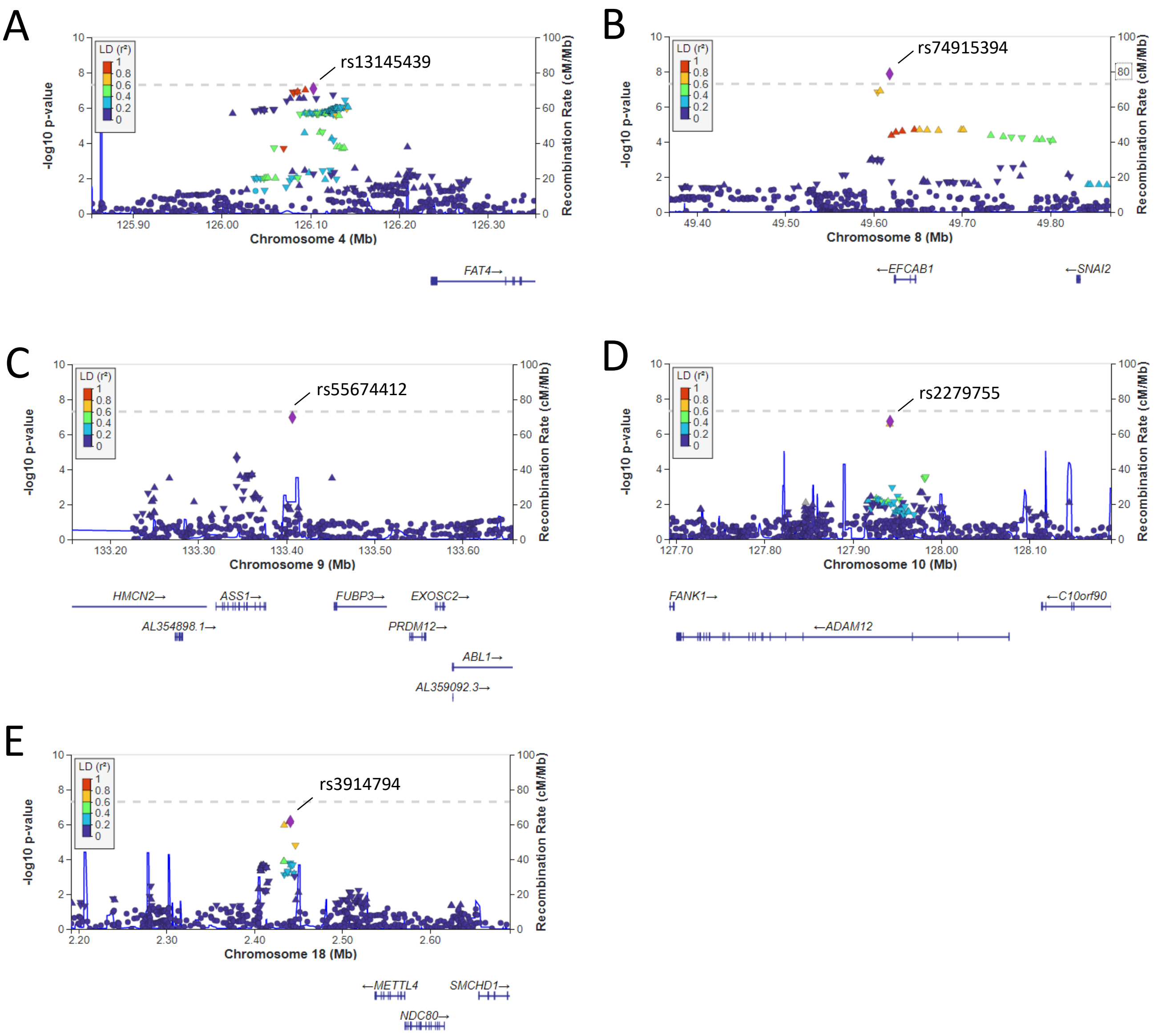

Figure S3. Locuszoom plots for genome-wide significant loci for absolute change in central subfield thickness. The *P* values of SNPs (shown as –log10 *P* values in y-axis) were plotted against their map positions (x-axis) with Locus Zoom (http://locuszoom.org). The selected SNP was in purple, and its linkage disequilibrium values (r^2) with nearby SNPs are indicated by different colors.

Figure S4

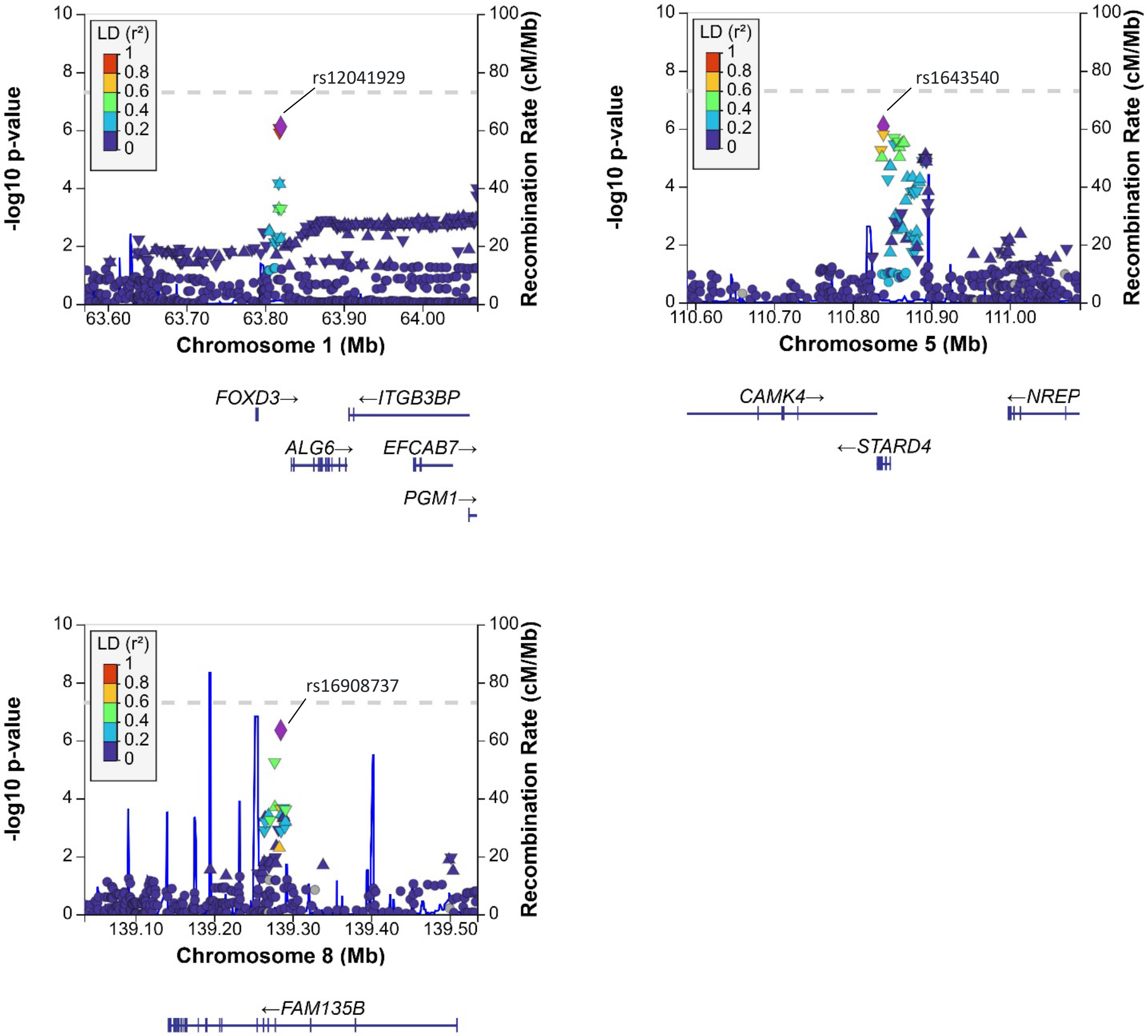

Figure S4. Locuszoom plots for genome-wide significant loci for relative change in central subfield thickness. The *P* values of SNPs (shown as –log10 *P* values in y-axis) were plotted against their map positions (x-axis) with Locus Zoom (http://locuszoom.org). The selected SNP is in purple.
